# Headaches during pregnancy and the risk of subsequent stroke

**DOI:** 10.1186/s10194-023-01689-9

**Published:** 2023-12-01

**Authors:** Ki-Woong Nam, Sungyeon Ha, Min-Jeong Oh, Kyungmi Oh, Chi Kyung Kim, Geum Joon Cho, Young Seo Kim, Cheryl D. Bushnell

**Affiliations:** 1 Department of Neurology, Seoul Metropolitan Government-Seoul National University Boramae Medical Center, Seoul, Korea; 2 Department of Neurology, Seoul National University College of Medicine, Seoul, Korea; 3 Graduate School of Statistics, Sungkyunkwan University, Seoul, Korea; 4 Department of Obstetrics and Gynecology, Korea University Guro Hospital, Korea University College of Medicine, 148 Gurodong-Ro, Guro-Gu, Seoul, 08308 Korea; 5 Department of Neurology, Korea University Guro Hospital, Korea University College of Medicine, 148 Gurodong-Ro, Guro-Gu, Seoul, 08308 Korea; 6 Department of Neurology, Korea University College of Medicine, Seoul, Korea; 7 Department of Neurology, College of Medicine, Hanyang University, Seoul, Korea; 8 Department of Neurology, Wake Forest School of Medicine, Winston-Salem, North Carolina United States

**Keywords:** Headache, Stroke, Pregnancy, Prognosis, Risk factor, Female stroke

## Abstract

**Background:**

Primary headache disorder is a known risk factor for stroke in women and usually improves during the first trimester of pregnancy. However, despite this, some women develop headaches during pregnancy (G-HA), and the effect of this headache on subsequent stroke is unknown. In this study, we evaluated the association between G-HA and stroke after delivery in women.

**Methods:**

Based on the Korean National Health Insurance Service database, we included women hospitalized for delivery between 2012 and 2013. G-HA was defined as a headache diagnosed during pregnancy. Primary outcome was any stroke that occurred during the observational periods from delivery to December 31, 2020. All diseases were identified based on data registered in the database using the International Classification of Disease-10^th^ Revision-Clinical Modification codes.

**Results:**

Of 906,187 pregnant women, G-HA was found in 56,813 (6.3%). During the observational periods, the G-HA ( +) group had a significantly higher risk of any stroke [adjusted hazard ratio (aHR) = 1.59, 95% confidence interval (CI): 1.30–1.95], ischemic stroke (aHR = 1.50, 95% CI: 1.12–2.01), hemorrhagic stroke (aHR = 1.63, 95% CI: 1.23–2.15), and intracerebral hemorrhage (aHR = 1.63, 95% CI: 1.19–2.23) than the G-HA (-) group. When analyzed considering the interaction with history of headache disorder, G-HA showed a significant association with hemorrhagic stroke, but lost its effect on ischemic stroke.

**Conclusions:**

We demonstrated that G-HA was associated with subsequent stroke occurrence after delivery. However, the relationship between G-HA and ischemic stroke is mitigated by a history of pre-pregnancy headache disorder.

**Supplementary Information:**

The online version contains supplementary material available at 10.1186/s10194-023-01689-9.

## Background

Headache is one of the most common symptoms that neurologists encounter in clinical practice, and it is common in young women [1–3]. Most of them are primary headache disorders and have a benign clinical course when appropriate treatment is given [4]. However, previous studies have shown that primary headache disorders can increase the risk of not only metabolic and cardiovascular diseases, but also cerebrovascular diseases [5–9]. Women with migraines had an increased risk of ischemic stroke of 1.7 to 2.0 times compared to women without migraines, and this tendency was evident in young women under the age of 45 years [10–13]. This association was the same in patients with nonspecific primary headache disorders, excluding migraines, with a 1.5-fold to 4.0-fold increase in the risk of stroke [14–16]. Therefore, identification of high-risk groups among women with primary headache disorders and appropriate management is necessary.

When a woman becomes pregnant, a number of physiological changes, including hormonal changes, occur in the body [17, 18]. As a result of these changes, 50%-80% of migraines and 30% of other primary headache disorders are improved in the first trimester of pregnancy [6, 17, 19]. However, in some women, the headaches do not improve, and 3%-6% of pregnant women develop a novel headache during pregnancy [20, 21]. Through several studies, migraine during pregnancy has been be associated with the occurrence of stroke during pregnancy [5, 6, 22]. However, these studies were mainly designed as cross-sectional studies, and there was no cohort study considering the variable of time. In addition, the effect of headaches experienced during pregnancy (i.e., gestational headache [G-HA]) on subsequent stroke after delivery is unknown yet.

In this study, we evaluated the association between G-HA and stroke after delivery using Korean National Health Insurance Service (NHIS) data. G-HA may be a new headache during pregnancy or may be a continuation of a previous headache disorder. Therefore, by analyzing the risk of ischemic or hemorrhagic stroke according to the presence/absence of G-HA and history of headache disorder (Hx-HA), we want to determine if women experiencing which types of headaches during pregnancy are at a higher risk for future stroke occurrences.

## Methods

All clinical data used in this study were obtained from the prospectively collected national health insurance claims database established by the Korean NHIS [23]. The Korean NHIS is a universal health insurance service that provides comprehensive medical care to up to 97% of Koreans [23]. The remaining 3% are low-income families, and since they are covered by the government-financed Medical Aid program operated by NHIS, virtually all citizens are included in the NHIS database [24]. The NHIS database contains claims data for overall medical service use, including individual diagnosis, treatment, procedure, hospitalization and discharge, and prescription records [24]. Among them, diagnosis-related information is recorded using the International Classification of Disease, 10th Revision, Clinical Modification (ICD-10-CM) codes. All data is encrypted and stored in the form of individual identification numbers to protect patients' personal information [23]. However, if a claim was made according to an appropriate procedure for research purposes, all claims belonging to the same patient can be obtained [23]. On the basis of this dataset, we evaluated the demographic and clinical factors necessary for this study.

This retrospective cohort study was approved by the Institutional Review Board (IRB) at Korea University Guro Hospital (IRB number: 2021GR0383). The requirement to obtain written informed consent form the study participants was waived due to the retrospective study design using anonymous information. All experiments were performed in accordance with the Declaration of Helsinki and relevant guidelines and regulations. All data and materials related to the article are included in the main text and supplemental materials.

### Study population and characteristics

We included women hospitalized for delivery between January 2012 and December 2013. Among them, 496 women with previous ischemic or hemorrhagic stroke and 2,837 women with missing values in the data were excluded. The remaining 906,187 were eligible for analyses.

The baseline characteristics prior to and during pregnancy across the demographic, clinical, and vascular risk factor areas were extracted from the database. These included age, hypertension, diabetes, previous functional disability, Hx-HA, gestational hypertension (include pre-eclampsia and eclampsia), gestational diabetes, and G-HA. Previous functional disability was defined as a ≥ 3 points in Charlson comorbidity index scores [25]. Hx-HA was defined as a primary headache disorder diagnosed before pregnancy and included migraine (ICD-10-CM code: G43.X), tension-type headache (ICD-10-CM code: G44.2), and other primary headache disorders (ICD-10-CM codes: G44.0, G44.1, R51.X). G-HA was defined as the occurrence of these headaches during pregnancy.

The primary outcome of this study was any stroke diagnosed during the identification period including postpartum and subsequent periods. Following the definition of previous studies, [26, 27] ischemic stroke was defined as when the following three conditions were satisfied: 1) have the corresponding diagnostic codes (ICD-10-CM code: I63.X), 2) perform brain imaging, and 3) be hospitalized for the corresponding stroke events. Hemorrhagic stroke was defined in the same way except using different diagnostic codes (ICD-10-CM codes: I60.X, I61.X, and I62.X). Because nontraumatic subdural hematoma and extradural hematoma are very rare in young age, we analyzed hemorrhagic stroke by dividing it into subtypes of intracerebral hemorrhage (ICH) and subarachnoid hemorrhage (SAH). Transient ischemic attack was excluded. To assess the outcomes, the participants were followed up to December 2020, and censored at the outcome events or at the end of the study period.

### Statistical analysis

Baseline characteristics and clinical outcomes between women with and without G-HA were evaluated. Continuous variables were presented as means ± standard deviations and categorical variables were presented as numbers and percentages, respectively. Differences between G-HA (-) group and G-HA ( +) group were analyzed using Student’s t-test or Mann–Whitney U-test for continuous variables and chi-squared tests of Fisher’s exact test for categorical variables.

The cumulative events of each stroke outcome were assessed using Kaplan–Meier analysis, and comparisons between G-HA (-) group and G-HA ( +) group were performed the log-rank test. The incidence rate was calculated based on the number of event outcomes, number of observed participants, and observation period of each participants. Considering the influence of confounding factors, multivariable cox regression analysis was performed to compare the differences in risk on the four types of stroke outcomes between women with and without G-HA. G-HA (-) group was set as a reference and the adjusted hazard ratio (aHR) and 95% confidence interval (CI) of G-HA ( +) group was calculated, indicating the relative risk difference between the two groups.

As mentioned earlier, G-HA may be an extension of Hx-HA, and there may be biological interactions between the two. To confirm this, we divided the entire cohort into four groups according to the presence or absence of G-HA and Hx-HA, and compared the cumulative incidence rates of stroke between them using Kaplan–Meier analysis and log rank test. In addition, the interaction term between G-HA and Hx-HA was created as a new variable, and the synergistic effect between G-HA and Hx-HA was confirmed in the four types of stroke outcomes by introducing and analyzing them together with multivariable cox regression analysis.

All statistical analyses were performed using SAS for Windows, version 9.4 (SAS Inc., Cary, NC, USA). In this study, all tests were two-sided, and variables with *P* < 0.05 were considered statistically significant.

## Results

Among a total of 906,187 women, 1,066 women were newly diagnosed with any stroke during the observation periods: 537 cases of ischemic stroke and 565 cases of hemorrhagic stroke. G-HA was diagnosed in 56,813 (6.3%) women. The median follow-up period for the cohort was 8.03 years.

The comparisons of baseline characteristics between women with and without G-HA are presented in Table 1. G-HA ( +) group had younger age, more frequent hypertension, diabetes, previous functional disability, Hx-HA, gestational hypertension, gestational diabetes, any stroke, ischemic stroke, hemorrhagic stroke, and ICH.

**Table 1:** Comparisons of baseline characteristics of population with and without gestational headache

	G-HA (-) (*n* = 849,374)	G-HA ( +) (*n* = 56,813)	*P* value
**Prior to pregnancy**			
Age, y [SD]	31.73 ± 4.09	31.25 ± 4.27	< 0.001
Hypertension, n (%)	4,495 (0.53)	574 (1.01)	< 0.001
Diabetes, n (%)	7,606 (0.9)	792 (1.39)	< 0.001
Previous functional disability, n (%)^a^	4,681 (0.55)	597 (1.05)	< 0.001
History of headache disorder, n (%)	28,364 (3.34)	5,465 (9.62)	< 0.001
Migraine	16,747 (1.97)	3,592 (6.32)	< 0.001
Tension-type headache	12,092 (1.42)	2,138 (3.76)	< 0.001
Other primary headaches	1,580 (0.19)	315 (0.55)	< 0.001
**During pregnancy**			
Gestational hypertension, n (%)^b^	12,537 (1.48)	1,279 (2.25)	< 0.001
Gestational diabetes, n (%)	49,699 (5.85)	3,253 (5.73)	0.217
**Clinical outcomes**			
Any stroke, n (%)	963 (0.11)	103 (0.18)	< 0.001
Ischemic stroke, n (%)	488 (0.06)	49 (0.09)	0.006
Hemorrhagic stroke, n (%)	509 (0.06)	56 (0.10)	< 0.001
Intracerebral hemorrhage, n (%)	395 (0.05)	44 (0.08)	0.001
Subarachnoid hemorrhage, n (%)	139 (0.02)	15 (0.03)	0.076

G-HA Gestational headache

^a^This variable was defined as ≥ 3 Charson comorbidity index score

^b^This variable included simple hypertension during pregnancy, pre-eclampsia, and eclampsia

**Fig. 1:**
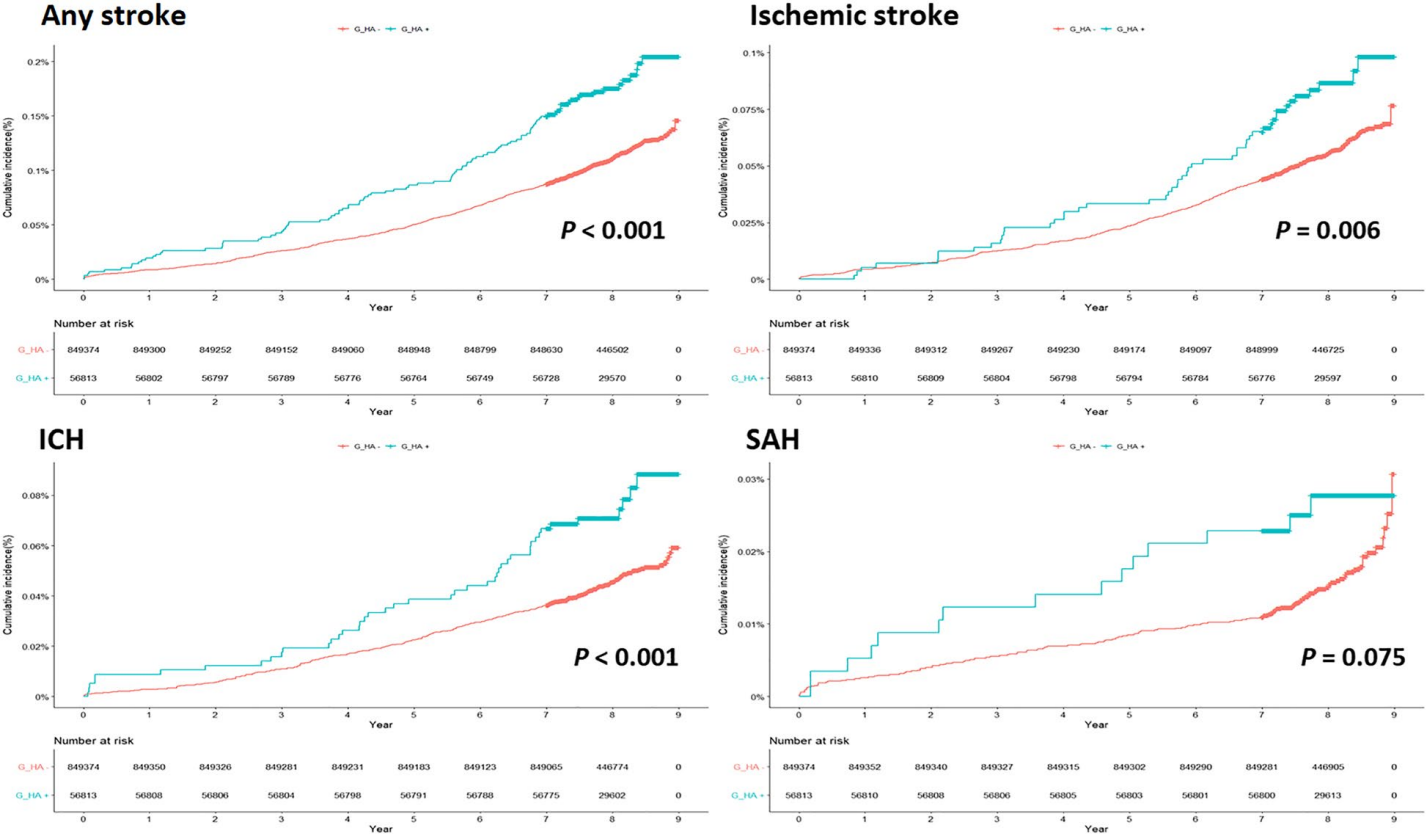
Cumulative incidence of four clinical outcomes in participants with and without gestational headaches. ICH = intracerebral hemorrhage, SAH = subarachnoid hemorrhage. Compared to women without gestational headache, women with gestational headache had a significantly higher risk of any stroke (**A**), ischemic stroke (**B**), and ICH (**C**). However, gestational headache showed no statistical correlation with SAH

**Fig. 2:**
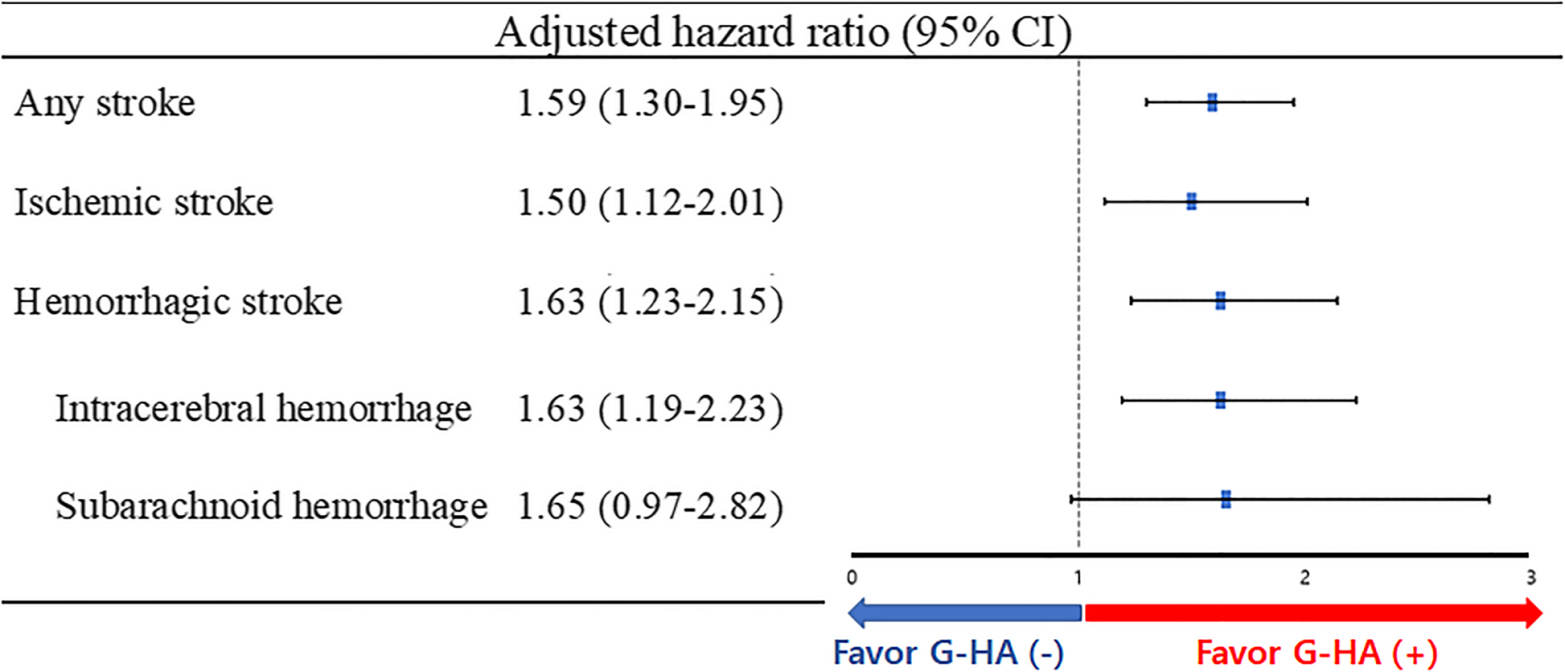
Adjusted hazard ratio of gestational headache in each of the four clinical outcomes: multivariable cox regression analysis^*^. ^*^Each outcome was adjusted for age, hypertension, diabetes, functional disability, history of headache, gestational hypertension, and gestational diabetes. Compared with the G-HA (-) group as the reference, the G-HA ( +) group had a significant risk increase for any stroke (59%), ischemic stroke (50%), hemorrhagic stroke (63%), and intracerebral hemorrhage (63%). However, there was no statistical difference between the G-HA ( +) group and the G-HA (-) group in the occurrence of subarachnoid hemorrhage

Figure 1 shows the cumulative incidence curves of the four stroke outcomes according to the presence or absence of G-HA. The G-HA ( +) group showed a higher cumulative incidence rate than the G-HA (-) group in all any stroke (*P* < 0.001), ischemic stroke (*P* = 0.006), hemorrhagic stroke (*P* < 0.001), and ICH (*P* = 0.001). However, the G-HA ( +) group showed no statistical association with the risk of SAH (P = 0.075). Figure 2 shows the adjusted hazard ratio (aHR) of G-HA by adjusting confounders for each stroke outcome in multivariable cox regression analysis. G-HA group was significantly associated with a higher risk of any stroke (aHR, 1.59; 95% CI, 1.30–1.95), ischemic stroke (aHR, 1.50; 95% CI, 1.12–2.01), hemorrhagic stroke (aHR, 1.63; 95% CI, 1.23–2.15), and ICH (aHR, 1.63; 95% CI, 1.19–2.23). Even after adjusting for confounders, G-HA showed no association with SAH (aHR, 1.65; 95% CI, 0.97–2.82).

**Fig. 3:**
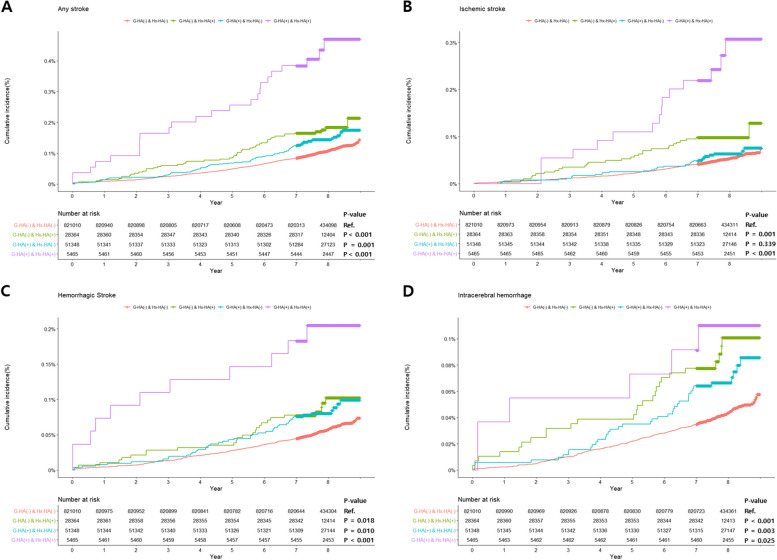
Cumulative incidence of four clinical outcomes between groups according to the history of headache disorders and gestational headaches. G-HA = gestational headache, Hx-HA = history of headache disorder. In a comparison between the four groups according to the presence or absence of G-HA and Hx-HA, the [G-HA ( +) & Hx-HA( +)] group showed the highest cumulative incidence rate in all types of stroke outcomes. The [G-HA ( +) & Hx-HA (-)] or the [G-HA (-) & Hx-HA ( +)] groups also showed higher cumulative incidence rates than the [G-HA (-) & Hx-HA (-)] group. However, in ischemic stroke, the [G-HA( +) & Hx-HA(-)] group showed no statistical difference from the [G-HA(-) & Hx-HA(-)] group

In our study population, 33,829 (3.7%) women had Hx-HA. In comparison between the groups according to the presence or absence of G-HA and Hx-HA, the [G-HA( +) & Hx-HA( +)] group showed the highest cumulative incidence rate compared to other groups in all types of stroke. The groups with either G-HA or Hx-HA also showed a higher cumulative incidence rate of stroke than the [G-HA(-) & Hx-HA(-)] group. However, in ischemic stroke, the [G-HA( +) & Hx-HA(-)] group showed no statistical difference from the [G-HA(-) & Hx-HA(-)] group (Fig. 3).

Based on this tendency, we analyzed the biological interaction of G-HA and Hx-HA on the occurrence of stroke after pregnancy. No statistical interaction between G-HA and Hx-HA was observed in any stroke, hemorrhagic stroke, or ICH, and both were independently associated with the risk of stroke. However, G-HA and Hx-HA showed a clear biological interaction in the occurrence of ischemic stroke after delivery, and when corrected for this, only Hx-HA was associated with ischemic stroke risk (Table 2). This trend was consistent even when the analysis excluded women who had experienced gestational hypertension (Additional file 1: Table S1).

**Table 2:** Hazard ratio of gestational headache in each of the four outcomes considering interaction with history of headache disorder

	Adjusted hazard ratio (95% confidence interval)^a^				
	Any stroke	Ischemic stroke	Hemorrhage stroke	ICH	SAH
Gestational headache	1.39 (1.11–1.75)	1.19 (0.84–1.69)	1.49 (1.10–2.02)	1.63 (1.17–2.28)	1.12 (0.57–2.20)
History of headache	1.61 (1.21–2.14)	1.83 (1.26–2.67)	1.53 (1.03–2.28)	1.99 (1.33–2.96)	1.35 (0.59–3.06)
Interaction term (G-HA x Hx-HA)	1.65 (0.97–2.83)	2.11 (1.04–4.32)	1.38 (0.64–2.98)	0.68 (0.26–1.75)	4.29 (1.15–16.03)

*G-HA* Gestational headache, Hx-HA History of headache disorder, ICH = intracerebral hemorrhage, SAH = subarachnoid hemorrhage. ^a^Each outcome was additionally adjusted for age, hypertension, diabetes, functional disability, gestational hypertension, and gestational diabetes

## Discussion

In this study, G-HA was associated with stroke after delivery in women. In addition, there seemed to be differences in the effects and interactions of G-HA and Hx-HA depending on the type of subsequent stroke. Therefore, by investigating the history of headache disorder before pregnancy and closely monitoring the G-HA occurrence during pregnancy, we expect to be able to identify high-risk groups for each type of stroke. This may help pregnant women establish a primary prevention plan of subsequent stroke after delivery.

We used Korean NHIS data in this study. The majority of Koreans use Korean NHIS because of its reasonable price and wide coverage [24]. In addition, since the physician has no choice but to enter the disease code and treatment code into the NHIS database after treatment in order to receive support for reimbursement of medical items, the NIHS database contains complete personal medical history until the occurrence of clinical events (e.g., stroke) or death of each Korean citizen [23, 24]. Thus, the Korean NHIS database minimized data loss and enabled complete follow-up during the observation period of our study population [23]. In other words, it is real-world data of pregnant women nationwide.

Our data showed a clear association between G-HA and stroke after delivery. However, we also found the relationship between G-HA and stroke occurrence should be interpreted differently depending on the stroke type. Hemorrhagic stroke showed a relatively clear association with G-HA. Both G-HA and Hx-HA were associated with a high risk of hemorrhagic stroke after delivery, but there was no interaction between the two on the occurrence of stroke. In other words, although the [G-HA ( +) & Hx-HA( +)] group showed the highest cumulative incidence rate of hemorrhagic stroke or ICH in Fig. 3, it means that G-HA was not related to Hx-HA. One might think that since secondary headaches accompanying hemorrhagic stroke were diagnosed as G-HA, a close correlation between the two was observed. However, in this study, we analyzed the association between headaches “during pregnancy” and subsequent stroke occurrence “after childbirth,” so there is a temporal difference between the two. Additionally, as shown in Additional file 1: Table S1, this hypothesis is rejected because there was no hemorrhagic stroke that occurred within 1 month after G-HA diagnosis. Initially, the authors assumed that the close association between G-HA and hemorrhagic stroke was the result of hemorrhagic complication immediately after delivery due to eclampsia/preeclampsia [28, 29]. However, even when further analysis excluded women with gestational hypertension, G-HA was still closely associated with hemorrhagic stroke (Additional file 2: Table S2). Additionally, among a total of 565 subsequent hemorrhagic stroke events, only 4 cases in the G-HA ( +) group occurred in the post-partum period (Additional file 3: Table S3). Therefore, we believe that the influence of this type of pathological mechanism is not likely to be significant. Primary headaches usually improve during the first trimester of pregnancy [6, 17]. Of course, even so, most cases of G-HA discovered during pregnancy will be primary headache disorder. However, some of these headaches may be secondary to organic lesions (e.g., vasculitis, cavernous malformation, and moyamoya disease) [30]. Therefore, in terms of hemorrhagic stroke after delivery, we may recommend brain magnetic resonance imaging to women who developed G-HA during pregnancy to identify organic lesions that may cause hemorrhagic stroke, regardless of previous history of primary headache disorders. Additionally, in our data, G-HA showed a close association with ICH, while it did not show statistical significance with SAH. Therefore, it can be also interpreted that G-HA may be associated with a long-standing hypertensive condition that persists even after delivery.

On the other hand, G-HA showed a limited association with subsequent ischemic stroke. Women with both G-HA and Hx-HA had a nearly fivefold higher risk of ischemic stroke compared with women with neither headache type. However, the association between the two disappeared when considering the interaction with Hx-HA. In addition, the [G-HA( +) & Hx-HA(-)] group did not show a statistically significant difference in risk of ischemic stroke from the [G-HA(-) & Hx-HA(-)] group. These results may indicate that the persistence of previous headache disorders even during pregnancy is more important than the occurrence of headaches during pregnancy itself in the occurrence of ischemic stroke after delivery. In fact, women with Hx-HA without G-HA also showed a significantly higher risk of ischemic stroke compared to the [G-HA(-) & Hx-HA(-)] group. History of primary headache disorders, especially migraine, is a well-known risk factor for stroke [31]. The vasospasm that occurs during migraine attack reduces cerebral blood flow, which can lead to an ischemic stroke [11–13]. In addition, in migraine patients, platelet aggregation is activated, various prothrombotic markers are elevated, and paradoxical embolism through the patent foramen ovale is prevalent [7, 10, 13, 15]. Usually, migraines improve in the first trimester of pregnancy, but if they persist during pregnancy, it can mean intractable migraines with severe vasospasm. Therefore, in terms of ischemic stroke, it seems important to establish a primary prevention plan by closely monitoring whether G-HA persists during pregnancy in pregnant women with a history of primary headache disorder.

In our data, the prevalence of stroke after delivery is very low at 0.12%. Considering that the median age of our study population is 31 years and the observation period is a maximum of 9 years, it can be seen that most of our data represent a young age stroke prevalence of < 40 years. Therefore, 0.12% is considered a reasonable prevalence. In fact, it is consistent with the Korea Stroke Epidemiology Report reported in 2018 and the data available from the National Statistical Office (http://kosis.kr). However, because these women are young, they will suffer from a disability for a very long time. Therefore, we think that paying attention to the primary prevention of stroke after delivery is sufficiently medically and sociologically meaningful.

Our study has several limitations. First, although it used the prospectively collected Korean NHIS database, this is a retrospective cohort study. Thus, our results confirm the association between G-HA and stroke after pregnancy, but do not imply a causality. Second, due to the retrospective nature of the study, we were unable to learn in detail about the headache symptoms of G-HA experienced by participants (e.g., migraine-like or tension-type-like). Third, the prevalence of history of headache disorder in our cohort was estimated to be lower than generally known. Of course, it is true that the prevalence of primary headache disorder has been reported to be lower in the Korean population than in the Caucasian population. However, since our cohort included medical records from 2011 to 2020 for women who gave birth between 2012 and 2013, the impact of only having a washout period of up to 2 years to evaluate past medical history may be more significant. This low prevalence may underestimate the influence of history of headache disorder. Fourth, due to the characteristics of Korea, most of the study population are Asians. Therefore, additional validation studies are needed to apply our results to other races. Fifth, because our study determines prevalence based on the disease code entered by the attending physician, people who had a previous history of headache disorder but did not visit the hospital may be classified as G-HA if they were first detected due to pregnancy. Finally, because most of the women included in the analysis were young, the accompanying vascular risk factors were small. Therefore, the possibility that the effect of G-HA on stroke was relatively overestimated should be considered because the effect of these comorbidities was small.

## Conclusion

We demonstrated that G-HA was closely related to stroke after delivery. Pregnant women should be socially protected, and preventing their subsequent stroke is an important issue not only for young women but also for their children. Based on our results, we believe that the taking a history of previous headache disorder (Hx-HA) and the regular examining for G-HA during pregnancy will be helpful in classifying high-risk groups for ischemic stroke and hemorrhagic stroke after delivery and devising a primary prevention strategy for each. However, our findings should be validated through subsequent prospective studies.

### Supplementary Information

**Additional file 1:** Frequency of subsequent stroke according to time after G-HA diagnosis in G-HA (+) group.

**Additional file 2:** Hazard ratio of gestational headache in each of the four outcomes considering interaction with history of headache disorder in participants without gestational hypertension.

**Additional file 3:** The prevalence of subsequent strokes in the post-partum period (up to 8wks) and thereafter.

## Data Availability

All data related to this study are included in the main text and the additional files.

## Abbreviations

G-HA: Gestational headache
NHIS: National health insurance service
Hx-HA: History of headache disorder
ICD-10-CM: The international classification of disease 10th revision clinical modification
ICH: Intracerebral hemorrhage
SAH: Subarachnoid hemorrhage
