# Supplementary material for: Headaches during pregnancy and the risk of subsequent stroke
Source: J Headache Pain. 2023 Dec 1;24(1):159. doi: 10.1186/s10194-023-01689-9 (PMC10691126; doi:10.1186/s10194-023-01689-9)
Supplement: Supplementary file 1 — Additional file 1: Frequency of subsequent stroke according to time after G-HA diagnosis in G-HA (+) group. [file 10194_2023_1689_MOESM1_ESM.docx]

**Additional file 1.** Frequency of subsequent stroke according to time after G-HA diagnosis in G-HA (+) group

| Time from diagnosis of G-HA | Any stroke  (n = 103) | Ischemic stroke  (n = 49) | Hemorrhage stroke  (n = 56) | ICH  (n = 44) | SAH  (n =15) |
| --- | --- | --- | --- | --- | --- |
| < 1m | 0 | 0 | 0 | 0 | 0 |
| 1m to < 6m | 2 | 0 | 2 | 1 | 0 |
| 6m to < 1y | 5 | 0 | 5 | 4 | 3 |
| ≥ 1y | 96 | 49 | 49 | 39 | 12 |

G-HA = gestational headache, ICH = intracerebral hemorrhage, SAH = subarachnoid hemorrhage
