# Supplementary material for: Headaches during pregnancy and the risk of subsequent stroke
Source: J Headache Pain. 2023 Dec 1;24(1):159. doi: 10.1186/s10194-023-01689-9 (PMC10691126; doi:10.1186/s10194-023-01689-9)
Supplement: Supplementary file 2 — Additional file 2: Hazard ratio of gestational headache in each of the four outcomes considering interaction with history of headache disorder in participants without gestational hypertension. [file 10194_2023_1689_MOESM2_ESM.docx]

**Additional file 2.** Hazard ratio of gestational headache in each of the four outcomes considering interaction with history of headache disorder in participants without gestational hypertension

|  | Adjusted hazard ratio (95% confidence interval)^*^ | | | | |
| --- | --- | --- | --- | --- | --- |
|  | Any stroke | Ischemic stroke | Hemorrhagic stroke | ICH | SAH |
| Gestational headache | 1.40 (1.11-1.77) | 1.13 (0.78-1.63) | 1.58 (1.16-2.15) | 1.71 (1.22-2.40) | 1.06 (0.52-2.16) |
| History of headache | 1.59 (1.19-2.13) | 1.80 (1.22-2.65) | 1.56 (1.04-2.33) | 2.05 (1.36-3.07) | 1.40 (0.62-3.19) |
| Interaction term (G-HA x Hx-HA) | 1.65 (0.95-2.88) | 2.32 (1.10-4.89) | 1.27 (0.57-2.82) | 0.70 (0.27-1.82) | 3.90 (0.98-15.60) |

G-HA = gestational headache, Hx-HA = history of headache, ICH = intracerebral hemorrhage, SAH = subarachnoid hemorrhage

^*^Each outcome was additionally adjusted for age, hypertension, diabetes, functional disability, and gestational diabetes
