# Supplementary material for: Headaches during pregnancy and the risk of subsequent stroke
Source: J Headache Pain. 2023 Dec 1;24(1):159. doi: 10.1186/s10194-023-01689-9 (PMC10691126; doi:10.1186/s10194-023-01689-9)
Supplement: Supplementary file 3 — Additional file 3: The prevalence of subsequent strokes in the post-partum period (up to 8wks) and thereafter. [file 10194_2023_1689_MOESM3_ESM.docx]

**Additional file 3.** The prevalence of subsequent strokes in the post-partum period (up to 8wks) and thereafter

|  | G-HA (-) | G-HA (+) | *P*-value |
| --- | --- | --- | --- |
| Any stroke |  |  |  |
| Post-partum | 30 (0.00) | 4 (0.01) | 0.186 |
| Subsequent | 933 (0.11) | 99 (0.17) | < 0.001 |
| Ischemic stroke |  |  |  |
| Post-partum | 15 (0.00) | 0 (0.00) | 0.317 |
| Subsequent | 473 (0.06) | 49 (0.09) | 0.003 |
| Hemorrhagic stroke |  |  |  |
| Post-partum | 16 (0.00) | 4 (0.01) | 0.011 |
| Subsequent | 493 (0.06) | 52 (0.09) | 0.002 |
| ICH |  |  |  |
| Post-partum | 10 (0.00) | 4 (0.01) | 0.012 |
| Subsequent | 385 (0.05) | 41 (0.07) | 0.004 |
| SAH |  |  |  |
| Post-partum | 11 (0.00) | 0 (0.00) | 0.391 |
| Subsequent | 128 (0.02) | 15 (0.03) | 0.037 |

G-HA = gestational headache, ICH = intracerebral hemorrhage, SAH = subarachnoid hemorrhage
